# Supplementary material for: Reconciling Mining with the Conservation of Cave Biodiversity: A Quantitative Baseline to Help Establish Conservation Priorities
Source: PLoS One. 2016 Dec 20;11(12):e0168348. doi: 10.1371/journal.pone.0168348 (PMC5173368; doi:10.1371/journal.pone.0168348)
Supplement: S1 Dataset — (ZIP) [file pone.0168348.s002.zip › Taxa/Serra Norte/SN_2013/N4E/Lista N4E-92.pdf]

CAVIDADE N4E-0092

Classe	Ordem	Fam/Outros	Gên/Outros	Espécie	S-U	U-S	Zon.
Annelida	Oligochaeta			jovem	1	3	E/P
Arachnida	Acari	(Oribatida)		jovem	X		P
Arachnida	Acari	(Parasitengonina)		sp.2		X	E/P
Arachnida	Acari	(Parasitengonina)		sp.4		X	P
Arachnida	Acari	Cunaxidae		sp.1	X		P
Arachnida	Acari	Dolichocybidae		sp.1		X	P
Arachnida	Acari	Ixodidae		jovem	X		P
Arachnida	Acari	Laelapidae		sp.1		X	P
Arachnida	Acari	Laelapidae		sp.3	X		P
Arachnida	Acari	Neothyridae	*Diplothyrus*	sp.1		X	E/P
Arachnida	Acari	Otopheidomenidae		sp.78	X		E
Arachnida	Acari	Prostigmata		sp.49	X		P
Arachnida	Acari	Rhagidiidae		sp.6		X	P
Arachnida	Acari	Trombiculidae		sp.39		X	P
Arachnida	Acari	Trombiculidae		sp.41	X		P
Arachnida	Amblypygi	Charinidae	*Charinus*	sp.1	1	1	P
Arachnida	Amblypygi	Charinidae	*Charinus*	sp.1	2	2	E/P
Arachnida	Amblypygi	Phrynidae	*Heterophrynus*	*longicornis*	2	2	E/P
Arachnida	Araneae	Barychelidae		jovem	3	2	P
Arachnida	Araneae	Corinnidae		jovem	1		P
Arachnida	Araneae	Ctenidae	*Ctenus*	sp.1	2	5	P
Arachnida	Araneae	Filistatidae		sp.1	X	X	E/P
Arachnida	Araneae	Ochyroceratidae	*Ochyrocera*	sp.3	X	X	E/P
Arachnida	Araneae	Ochyroceratidae	*Speocera*	sp.1	X		P
Arachnida	Araneae	Pholcidae	*Leptopholcus*	sp.1	X		E
Arachnida	Araneae	Pholcidae	*Mesabolivar*	aff. *cambridgei*	X		P
Arachnida	Araneae	Pholcidae	*Mesabolivar*	*aurantiacus*	X	X	E/P
Arachnida	Araneae	Pholcidae	*Mesabolivar*	sp.1	X		E/P
Arachnida	Araneae	Pholcidae	Ninetinae	sp.1	X	X	P
Arachnida	Araneae	Salticidae		jovem	X	X	E/P
Arachnida	Araneae	Scytodidae	*Scytodes*	jovem		1	P
Arachnida	Araneae	Theraphosidae	aff. *Holothele*	sp.1	4	4	E/P
Arachnida	Araneae	Theridiidae	*Theridion*	sp.1	X	X	P
Arachnida	Araneae	Theridiosomatidae	*Plato*	sp.1	X	X	E/P
Arachnida	Opiliones	Escadabiidae		sp.2	X	X	P
Arachnida	Opiliones	Stygnidae	*Paraphareus*	sp.1		X	P
Arachnida	Palpigradi	Eukoeneniidae	*Eukoenenia*	sp.1		X	E
Arachnida	Pseudoscorpiones	Bochicidae		sp.1	X		P
Arachnida	Pseudoscorpiones	Chthoniidae	*Pseudochthonius*	sp.1	X	X	P
Arachnida	Pseudoscorpiones	Olpiidae		jovem	X	X	E
Arachnida	Schizomida	Hubbardiidae	aff. *Rowlandius*	sp.1		X	P
Chilopoda	Scolopendromorpha	Scolopendridae		jovem		1	E
Chilopoda	Scolopendromorpha	Scolopocryptopidae	*Dinocryptops*	*miersii*	1		E
Chilopoda	Scutigeromorpha	Pselliodidae		jovem		1	E
Diplopoda	Polydesmida	Chelodesmidae		jovem	2	19	P
Diplopoda	Polydesmida	Chelodesmidae		sp.3	3	19	P
Diplopoda	Polydesmida	Cyrtodesmidae		sp.1	X		E
Diplopoda	Polydesmida	Pyrgodesmidae		sp.1	X	X	E/P
Entognatha	Collembola	Cyphoderidae		sp.1	X		P

Entognatha	Collembola	Entomobryidae		sp.4		X	E
Entognatha	Collembola	Entomobryidae		sp.6	X		E
Entognatha	Collembola	Isotomidae		sp.1	X	X	E/P
Entognatha	Collembola	Paronellidae		sp.1	X	X	E/P
Entognatha	Collembola	Paronellidae		sp.4		X	P
Entognatha	Collembola	Sminthuroidea		sp.2	X	X	P
Entognatha	Diplura	Campodeidae		sp.1	X	X	E/P
Entognatha	Diplura	Projapygidae		sp.1	X	X	P
Entognatha	Diplura	Projapygidae		sp.2	X		E
Insecta	Archaeognatha	Meinertellidae		sp.1		X	E
Insecta	Blattodea	Blattellidae		jovem	3	2	E/P
Insecta	Blattodea	Blattidae		sp.5	2	6	E
Insecta	Blattodea	Polyphagidae		sp.1		2	P
Insecta	Coleoptera	Carabidae		sp.3		X	E
Insecta	Coleoptera	Staphylinidae		sp.59	X		P
Insecta	Coleoptera	Staphylinidae		sp.60	X		E
Insecta	Diptera	Cecidomyiidae	Cecidomyiinae	sp.	X	X	E/P
Insecta	Diptera	Culicidae	*Culex*	sp.	X	X	E/P
Insecta	Diptera	Dolichopodidae		sp.	X		E
Insecta	Diptera	Drosophilidae	*Drosophila*	*eleonorae*		X	P
Insecta	Diptera	Limoniidae		sp.		X	E
Insecta	Diptera	Phoridae	Metopininae	sp.	X		P
Insecta	Diptera	Psychodidae	Phlebotominae	sp.	X	X	E/P
Insecta	Diptera	Psychodidae	*Telmatoscopus*	sp.		X	P
Insecta	Diptera	Tipulidae	Tipulinae	sp.	X		P
Insecta	Heteroptera	Cydnidae		sp.	X	X	E/P
Insecta	Heteroptera	Dipsocoroidea		sp.	X		P
Insecta	Heteroptera	Reduviidae	Emesinae	sp.6	1	3	E/P
Insecta	Heteroptera	Reduviidae	Reduviinae	sp.	4	5	E/P
Insecta	Heteroptera	Reduviidae	Triatominae	sp.	3		P
Insecta	Homoptera	Cixiidae		jovem	X	X	P
Insecta	Hymenoptera	Formicidae	*Camponotus*	sp.1	X	X	E/P
Insecta	Hymenoptera	Formicidae	*Camponotus*	sp.2	X		P
Insecta	Hymenoptera	Formicidae	*Dolichoderus*	aff. *bispinosus*	X	X	E/P
Insecta	Hymenoptera	Formicidae	*Pachycondyla*	sp.1		X	
Insecta	Hymenoptera	Formicidae	*Pachycondyla*	sp.2	X		P
Insecta	Hymenoptera	Formicidae	*Paratrechina*	sp.2	X		P
Insecta	Hymenoptera	Formicidae	*Pheidole*	sp.2	X		E
Insecta	Hymenoptera	Formicidae	*Solenopsis*	sp.1	X	X	P
Insecta	Hymenoptera	Vespidae		sp.1	X		E
Insecta	Isoptera	Rhinotermitidae	*Heterotermes*	sp.	X		E
Insecta	Isoptera	Termitidae	*Nasutitermes*	sp.	X	X	E/P
Insecta	Lepidoptera	Noctuoidea		sp.1	1	1	E
Insecta	Lepidoptera	Tineoidea		sp.1	X	X	E
Insecta	Neuroptera	Myrmeleontidae		jovem	X	X	E
Insecta	Orthoptera	Gryllidae	Eneopterinae	jovem	1		P
Insecta	Orthoptera	Phalangopsidae	*Paraclodes*	sp.	2		E
Insecta	Orthoptera	Phalangopsidae	*Phalangopsis*	sp.	102	150	E/P
Insecta	Psocoptera	Amphipsocidae	*Calocaecilius*	sp.1		X	E
Insecta	Psocoptera	Archipsocidae	*Archipsocus*	sp.2		X	E
Insecta	Psocoptera	Epipsocidae	*Epipsocus*	sp.2	X		P
Insecta	Psocoptera	Psyllipsocidae	*Psyllipsocus*	sp.1	X	X	E

Insecta	Psocoptera	Psyllipsocidae	*Psyllipsocus*	sp.2	X		P
Insecta	Siphonaptera	Leptopsyllidae		sp.1		X	P
Insecta	Thysanura	Nicoletiidae		sp.1		X	P
Gastropoda	Pulmonata	Bulimulidae	*Naesiotus*	sp.	X	X	P
Gastropoda	Pulmonata	Systrophiidae	*Happia*	sp.	X	X	E/P
Malacostraca	Isopoda	Philosciidae		sp.1	X	X	P
Symphyla		Scutigerellidae		sp.		X	E
Amphibia	Anura	Strabomantidae	*Pristimantis*	cf. *fenestratus*	6		P
Mammalia	Chiroptera	Emballonuridae	*Peropteryx*	*kappleri*	3	10	E/P
Mammalia	Chiroptera	Furipteridae	*Furipterus*	*horrens*		8	E/P
Mammalia	Chiroptera	Phyllostomidae	*Anoura*	*caudifer*	4		P
Mammalia	Chiroptera	Phyllostomidae	*Anoura*	*geoffroyi*	100		P
Mammalia	Chiroptera	Phyllostomidae	*Carollia*	*perspicillata*		15	E/P
